# Modulation of functional activity and connectivity by acupuncture in patients with Alzheimer disease as measured by resting-state fMRI

**DOI:** 10.1371/journal.pone.0196933

**Published:** 2018-05-15

**Authors:** Weimin Zheng, Zhuangzhi Su, Xingyun Liu, Hao Zhang, Ying Han, Haiqing Song, Jie Lu, Kuncheng Li, Zhiqun Wang

**Affiliations:** 1 Department of Radiology, Dongfang Hospital, Beijing University of Chinese Medicine, Beijing, China; 2 Department of Radiology, Xuanwu Hospital of Capital Medical University, Beijing, China; 3 Department of Neurology, Xuanwu Hospital of Capital Medical University, Beijing, China; Banner Alzheimer's Institute, UNITED STATES

## Abstract

Acupuncture has been used in the therapy of Alzheimer disease (AD); however, its neural mechanisms are still unclear. The aim of this study is to examine the effect of acupuncture on the functional connectivity in AD by using resting-state functional magnetic resonance imaging (rs-fMRI). Twenty-eight subjects (14 AD and 14 normal controls) participated in this study. The rs-fMRI data were acquired before and after acupuncture stimulation at the acupoints of Tai chong (Liv3) and Hegu (LI4). During the baseline resting state, by using the amplitude of low-frequency fluctuations (ALFF), we found a significantly decreased or increased ALFF in the AD patients relative to the controls. These regions were located in the right superior frontal gyrus (SFG), left postcentral gyrus, subgenual cingulate cortex (SCC), right middle cingulate cortex (MCC), right inferior frontal gyrus (IFG), right hippocampus and the right inferior temporal gyrus (ITG). Then, we selected these brain regions as seeds to investigate whether regional activity and functional connectivity could be modulated by acupuncture in the AD patients. When compared to the pre-acupuncture stage, several of the above regions showed an increased or decreased ALFF after acupuncture in the AD patients. In addition, the functional connectivity between the hippocampus and the precentral gyrus showed enhancement after acupuncture in the AD patients. Finally, there were close correlations between the functional activity, connectivity and clinical performance in the AD patients. The current study confirmed that acupuncture at Tai chong (Liv3) and He gu (LI4) can modulate functional activity and connectivity of specific cognition-related regions in AD patients.

## Introduction

Alzheimer disease (AD) is a progressive neurodegenerative disease that manifests with memory deficits and cognitive decline [1, 2]. So far, there are over 24 million people suffering from AD all over the world, and the population may double to an estimated 42 million by 2020 [3]. Currently, there is no effective treatment for the disease, which stimulates researchers to find new ways of treating AD. As a classic treatment in traditional Chinese medicine (TCM), acupuncture has increasingly attracted attention, as it is thought to be effective in slowing the progression of AD. However, the neural mechanism underlying the effects of acupuncture is not very clear at present.

Resting-state functional magnetic resonance imaging (rs-fMRI) is a highly promising non-invasive imaging technique that is applied in the study of many neuropsychiatric diseases [4–7]. By measuring low frequency (0.01–0.08 Hz) fluctuations in the blood oxygenation level-dependent (BOLD) signal, rs-fMRI can reflect spontaneous brain activity and functional connectivity in vivo [8–11]. Several rs-fMRI studies have demonstrated that AD presents with disrupted functional characteristics at different levels, including in the amplitude of low-frequency fluctuations (ALFF) [12], regional homogeneity [13] and inter-regional functional connectivity [8, 14–17]. On the other hand, accumulating rs-fMRI evidence has suggested that the intrinsic brain functional architecture can be modulated by acupuncture [18–22]. Thus, we speculated that acupuncture can modulate or enhance resting-state functional activity and connectivity of cognition-related regions to achieve the effective treatment of AD.

At present, only a few rs-fMRI studies have investigated acupuncture’s effects on AD patients. By using regional brain activity analysis, two previous studies focused on the spontaneous brain activity in AD patients and found that some brain regions, such as the temporal and parietal lobe regions, were activated by acupuncture [23, 24]. By using the hippocampus as a seed region, one recent fMRI study found that AD patients showed increased connectivity in most of the hippocampus-associated regions after acupuncture [25]. Another study found that acupuncture stimulation could modulate default mode network (DMN) activity in AD patients [26]. These AD studies explored the effect of acupuncture on either regional spontaneous activity or on a specific brain network. However, until now, there have been no investigations exploring the effect of acupuncture on AD by combining regional activity analysis and seed-based functional connectivity analysis.

In this study, we first explored the regional brain activity changes of AD patients at a baseline resting state. Here, we used the ALFF method, which reflects regional spontaneous cerebral neural activity by calculating the square root of the power spectrum of the BOLD signals in a low-frequency range (usually 0.01–0.08 Hz) [27]. Second, we selected the regions that were significantly changed in the ALFF as seeds and calculated the regional activity and functional connectivity (FC) of these regions using a seed-based approach. We sought to investigate whether regional activity and interregional connectivity could be modulated by acupuncture in AD patients compared to healthy controls. In the current study, we chose Tai chong and He gu, which are collectively named the Si Guan (the four gates) point. The combined use of these two acupoints can harmonize yin and yang, regulate qi and blood, and finally can improve the cognitive ability of AD patients. Therefore, we chose these two acupoints. Finally, we explored the correlation between functional brain activity and connectivity and cognitive performances. Based on previous studies, we hypothesized that (1) AD patients would show ALFF changes in specific cognition-related brain regions and that (2) acupuncture can enhance the regional activity and functional connectivity of specific cognition-related brain regions in AD patients.

## Materials and methods

### Subjects

In our study, we recruited 28 subjects who were are all right-handed, including 14 AD patients and 14 age-, gender- and education-matched healthy controls (HCs). The AD patients were recruited from Xuanwu Hospital, Beijing, China, and the HCs were recruited from the local community by advertisements. The collecting time range is from June 2011 to February 2012. All the participants gave written informed consent. This study was approved by the Medical Research Ethics Committee of Xuanwu Hospital. The authors had no access to information that could identify individual participants during or after data collection. The clinical and demographic information for all the participants are shown in **Table 1**.

**Table 1:** Characteristics of the AD patients and healthy controls.

Characteristics	AD	HC	*P* value
N (M/F)	14(6/8)	14(6/8)	-
Age, years	66.92±8.91	66.07±5.78	0.86*
Education, years	8.71±3.03	11.79±4.13	0.61*
MMSE	16.29±4.88	28.14±1.30	<0.01*
AVLT(immediate)	11.21±4.07	25.71±5.03	<0.01*
AVLT(delayed)	2.29±1.58	10.57±2.77	<0.01*
AVLT(recognition)	2.93±1.83	12.57±1.99	<0.01*
CDR	1–2	0	-
MoCA	13.29±4.86	27.36±1.87	<0.01*

MMSE, Mini-Mental State Examination; Plus-minus values are means ± S.D. AVLT, Auditory verbal learning test; immediate, immediate recall of learning verbal; delayed; delayed recall of learning verbal; recognition, recognition of learning verbal; CDR, clinical dementia rate.; MoCA, Montreal Cognitive Assessment.

*The P values were obtained by one-way analysis of variance tests.

All the participants were given an extensive battery of neuropsychological assessments. The measurement scales were based on the Diagnostic and Statistical Manual of Mental Disorders 4th Edition criteria for dementia, the National Institute of Neurological and Communicative Disorders and the Stroke/Alzheimer Disease and Related Disorders Association (NINCDSADRDA) [28,29]. The HCs recruited for our study should have had no neurological diseases and no cognitive dysfunction.

### Data acquisition

Each of the subjects underwent an MRI scan in a SIEMENS verio 3-Tesla scanner (Siemens, Erlangen, Germany) and was instructed to remain still, keep their eyes closed and to think of nothing. The main MRI sequence and parameters were as follows: the functional MRI was acquired axially using echo planar imaging (EPI), repetition time (TR)/echo time (TE)/flip angle (FA) = 2000 ms/40 ms/90°, field of view (FOV) = 24 cm, image matrix = 64×64, slice number = 33, thickness = 3 mm, gap = 1 mm, and bandwidth = 2232 Hz/pixel.

**Fig 1:**
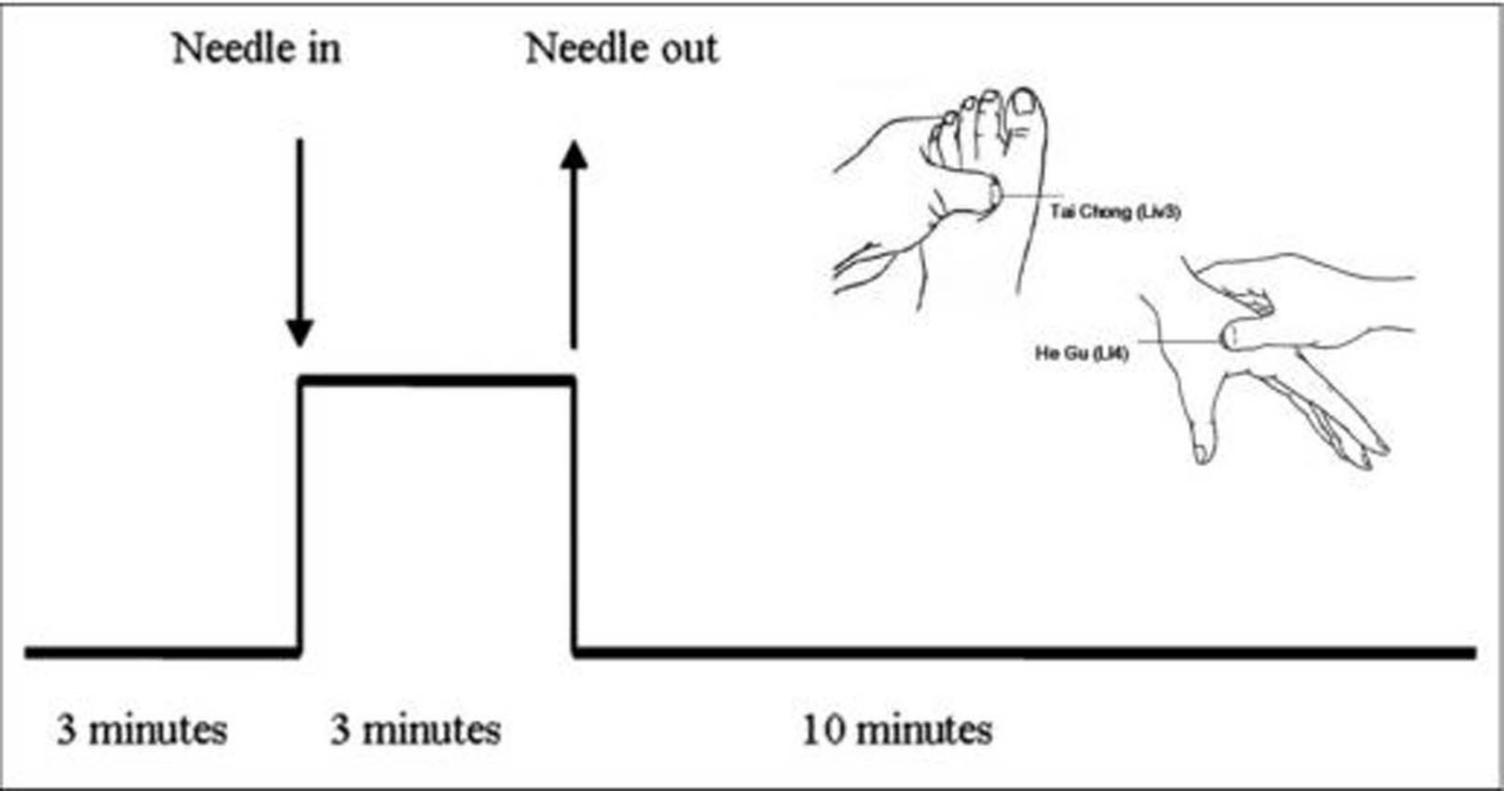
Protocols for acupuncture.

In the current study, we used a single-block experimental design, which is shown in **Fig 1**. During the initial 3 minutes, we acquired baseline resting-state data; then, we performed 3 minutes of acupuncture stimulation at four acupoints of the human body (Tai chong on the dorsum of the left and right feet and He gu on the dorsum of the left and right hands) using a silver needle that was 0.30 mm in diameter and 25 mm long. When the acupuncture stimulation finished, another 10 minutes of resting-state fMRI scans were acquired.

### Imaging preprocess

The data preprocessing was performed using the Statistical Parametric Mapping (SPM8) (http://www.fil.ion.ucl.ac.uk/spm/) and the Data Processing Assistant for rs-fMRI (DPARSFA; http://www.restfmri.net) [30] toolkits. First, the data were converted from the EPI DICOM to the NIFTI format, and the first 10 volumes were removed. Then, slice timing correction and realign were performed; during the realign, the subjects whose head motion exceeded 2.5 mm in translation or 2.5° in rotation in any direction were excluded. Next, the realigned volumes were spatially standardized to the MNI (Montreal Neurological Institute) space by normalizing them with the EPI template via their corresponding mean image. After normalization, the functional images were smoothed with a Gaussian kernel of 4 mm full width at half-maximum (FWHM). Finally, we regressed out the confounding factors, including six motion parameters, linear drift, the white matter signal and the cerebrospinal fluid (CSF) signal.

### ALFF and functional connectivity analysis

The ALFF analysis was performed using the DPARSFA (http://www.restfmri.net). After preprocessing, to reduce the effect of low-frequency drift and high-frequency physiological noise, bandpass filtering (0.01–0.08 Hz) was performed.

To investigate the functional alterations in the AD patients, we further performed seed-based functional connectivity analysis. As reported below, significant ALFF abnormalities in the patients with AD were demonstrated in seven brain regions, and these seven regions were used as seeds for the FC analysis.

### Statistical analysis

To assess the between-group differences (between the AD patients and the HCs) of the ALFF at the baseline resting state, two-sample t tests were performed with age, gender, education level and the mean FDR being treated as covariates. The significance threshold was set to P < 0.001 with topo-FDR correction.

Then, the regions that were significantly changed in terms of the ALFF were selected as seeds, and a paired-samples t test analysis was performed to investigate the differences among the selected regions between the pre-acupuncture and post-acupuncture stages in the AD patients.

We defined the functional ROIs according to the activated clusters. Our aim was to determine if the effect of acupuncture could ameliorate the functional pathways.

First, we extracted the z values for the pre-acupuncture and post-acupuncture stages for the AD patients. Then, an independent-samples t-test was run for each of the ROIs (pre-acupuncture versus post-acupuncture).

Finally, a partial correlation analysis was performed to explore the associations of the clinical variables with the functional activity and connectivity in the AD patients, with age, gender and education being used as nuisance covariates (P<0.05).

## Results

### Clinical and neuropsychological examination

**Table 1 showed** the clinical data of the subjects. No significant differences in age and gender was found between the AD and HC groups (both P > 0.1), but the AD group exhibited significantly lower Montreal Cognitive Assessment (MoCA), Mini-mental State Examination (MMSE), Auditory Verbal Learning Test (AVLT) and Clinical Dementia Rate (CDR) scores than those of the HC group (P < 0.001).

### Between group ALFF analysis in the resting state

**Fig 2:**
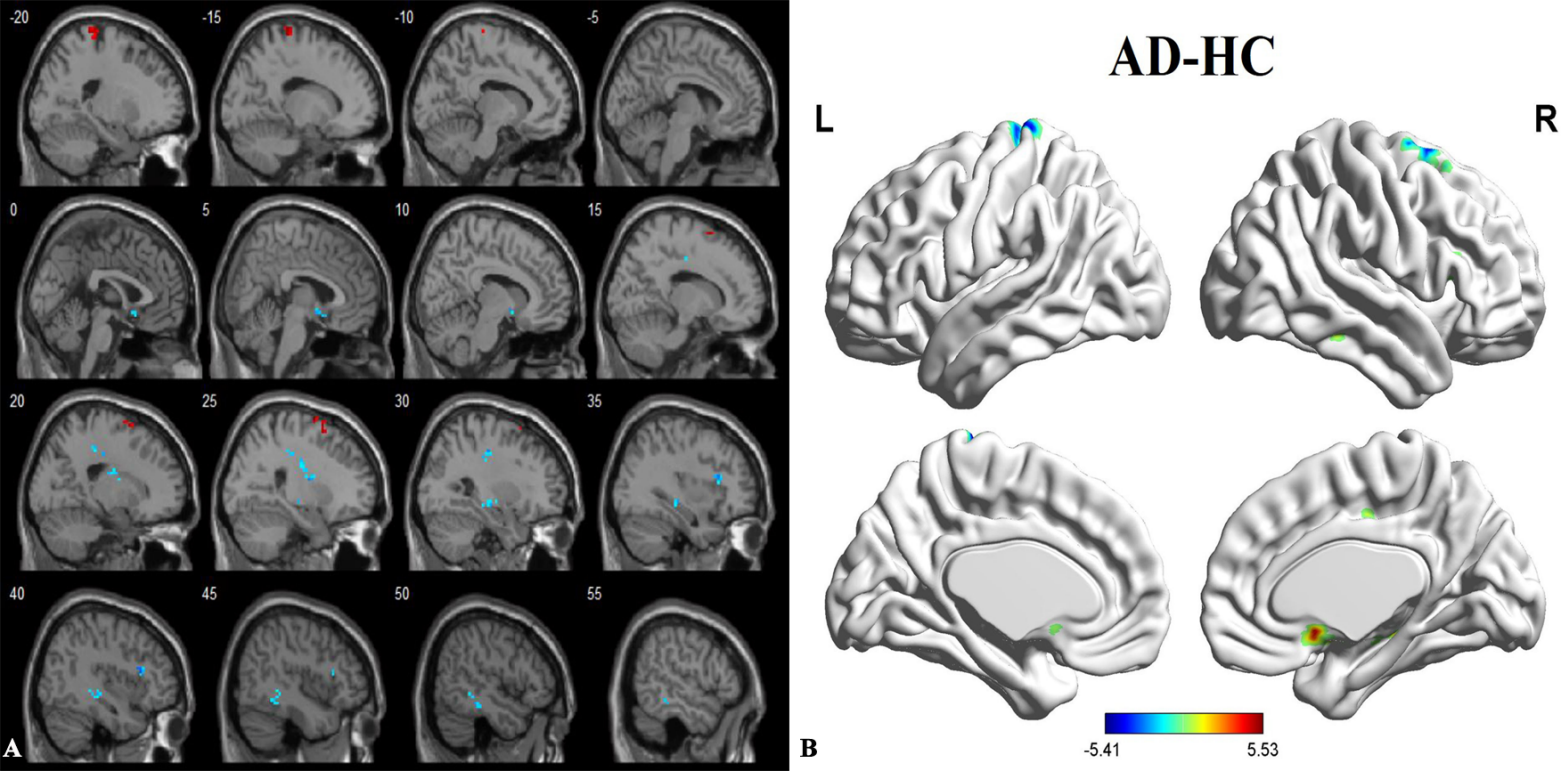
**(A and B).** Between-group differences in AD and HC before acupuncture, based on a 2-sample t test with age, gender, education level and mean FDR as covariates. The threshold was set to P < 0.001 with topo FDR corrected. The color scale represents t values. Cold color represents decreased ALFF in AD patients compared to HC. AD, Alzheimer’s disease; HC, healthy control; ALFF, amplitude of low-frequency fluctuations.

Compared to the healthy controls, the patients with AD showed a significantly decreased ALFF in the right superior frontal gyrus (SFG) and the left postcentral gyrus. A significantly increased ALFF was found in the subgenual cingulate cortex (SCC), right middle cingulate cortex (MCC), right inferior frontal gyrus (IFG), right hippocampus and right inferior temporal gyrus (ITG). The peak voxels within those significantly different clusters are shown in **Fig 2 and Table 2**.

**Table 2:** Regions showing decreased and increased brain activities in AD patients comparing to HCs before acupuncture.

Brain regions	Cluster voxels	MNI coordinates(mm)			Maximum Z
		x	y	z	
SCC	19	3	12	-9	4.98
MCC_R	22	27	-30	39	5.23
IFG_R	19	39	21	15	5.53
SFG_R	26	24	0	72	-5.41
Hippocampus_R	31	27	-12	-6	4.87
Postcentral_L	34	-15	-30	78	-4.93
ITG_R	25	51	-36	-15	4.71

P < 0.001 with topo FDR corrected. AD, Alzheimer’s disease; HC, healthy control; SCC, subgenual cingulate cortex; MCC, middle cingulate cortex; IFG, inferior temporal gyrus; SFG, superior frontal gyrus; ITG, inferior temporal gyrus.

### ALFF changes between post and pre-acupuncture in the AD patients

**Fig 3:**
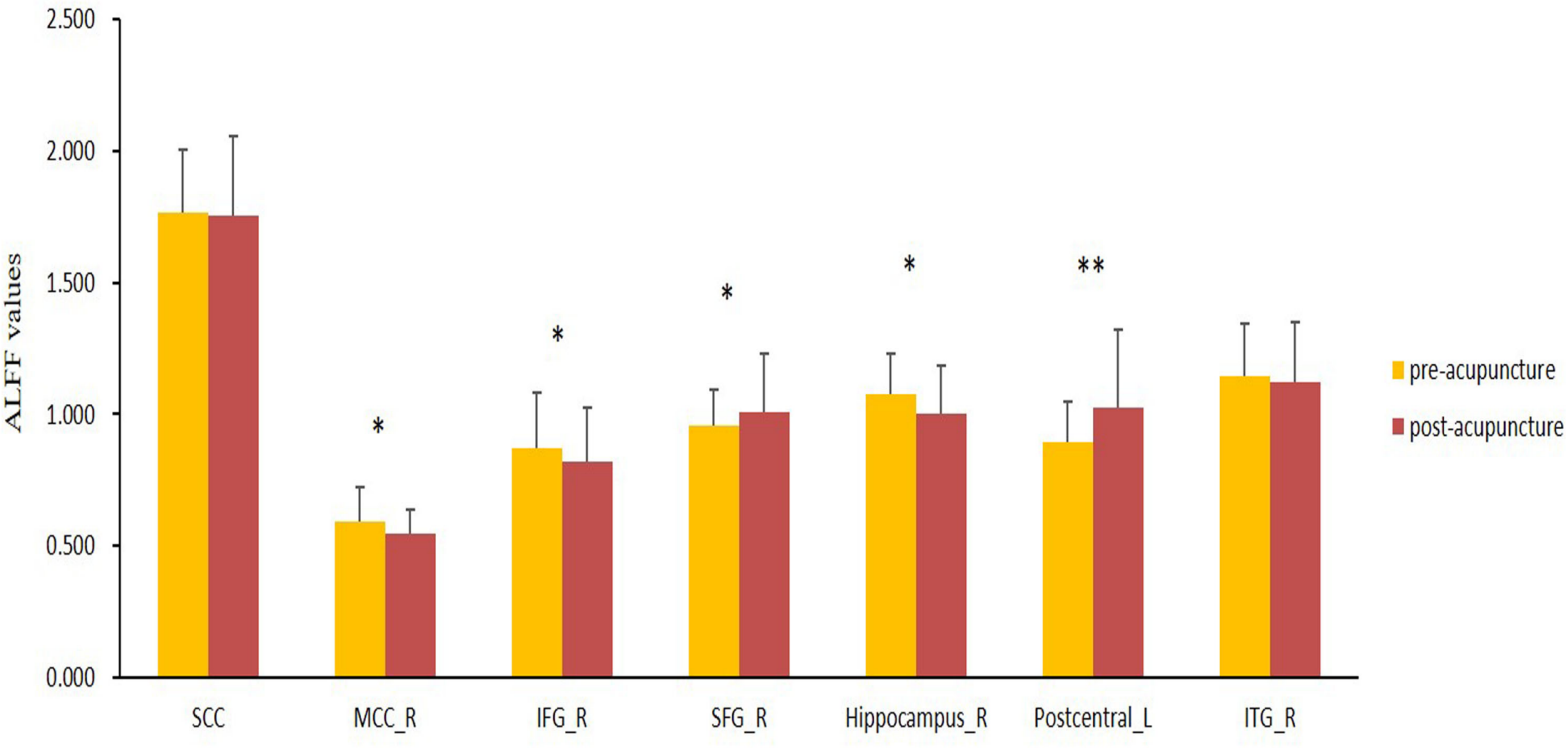
ALFF changes between post-acupuncture and pre-acupuncture in the AD patients, using the paired-samples T test analysis. SCC, subgenual cingulate cortex; MCC, middle cingulate cortex; IFG, inferior frontal gyrus; SFG, superior frontal gyrus; ITG, inferior temporal gyrus; **represents significantly changed ALFF in the AD patients when comparing post acupuncture with pre acupuncture (P<0.05); *represents slightly changed ALFF in the AD patients when comparing post acupuncture with pre acupuncture (P>0.05).

To explore the functional activity changes in the AD patients following acupuncture, we selected the regions that were significantly changed in terms of the ALFF as seeds, and then a paired-samples t test analysis was performed. **Fig 3** shows the significantly increased ALFF in the left postcentral gyrus in the AD patients when comparing the post-acupuncture with the pre-acupuncture stage (P<0.05). The right SFG also showed an increased ALFF after acupuncture although the statistical result did not reach the significance threshold. The right IFG, right hippocampus and MCC showed decreased ALFF after acupuncture, while the right ITG and SCC showed no significant different between the post-acupuncture and pre-acupuncture stages.

### Functional connectivity between post and pre-acupuncture in the AD patients

**Fig 4:**
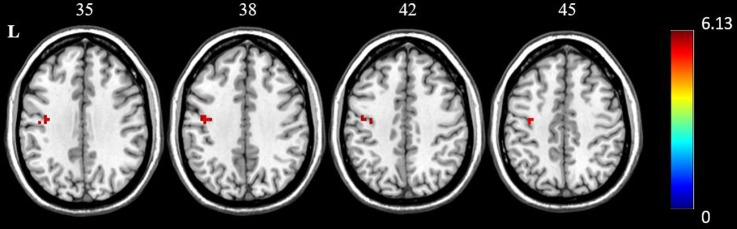
The FC differences between pre-acupuncture and post-acupuncture in AD patients (P<0.001, with topoFDR corrected). Regions which were significantly changed in ALFF were selected as seeds to perform the seed-based interregional correlation analysis. Increased connectivity between the right hippocampus and the left precentral gyrus was found in the AD patients when comparing post-acupuncture with pre-acupuncture.

To investigate the functional alterations in the AD patients following acupuncture, a seed-based interregional correlation analysis was performed. We selected the regions that were significantly ALFF differences of the two groups as seeds. **Fig 4 and Table 3** showed the increased connectivity between the right hippocampus and the left precentral gyrus in the AD patients when comparing the post-acupuncture with the pre-acupuncture stages.

**Table 3:** Region showing increased hippocampal connectivity in AD subjects after acupuncture comparing to before acupuncture.

ROI	Brain region	Cluster voxels	MNI coordinates(mm)			Maximum Z
			x	y	z	
Hippocampus_R	L.Precentral	22	-33	-12	45	6.12

P < 0.001 with topo FDR corrected. AD, Alzheimer’s disease.

### Correlations between functional activity, connectivity and clinical performance in the AD patients

**Fig 5:**
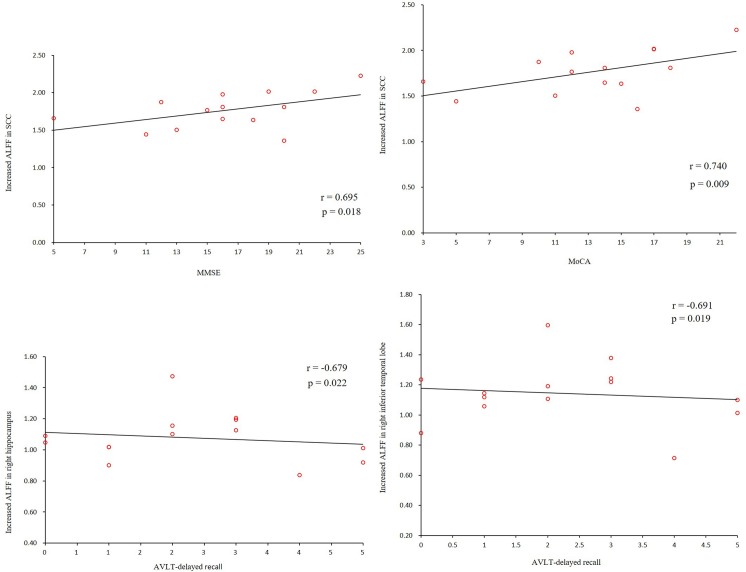
Correlations of clinical variables and ALFF values in the AD patients. MMSE, MoCA scores showed positive correlations with ALFF values of the SCC. Negative correlations were shown between AVLT scores and ALFF values of the hippocampus and the right ITG (P<0.05). ALFF, amplitude of low-frequency fluctuations; AD, Alzheimer’s disease; MMSE, Mini-Mental State Examination; MoCA, Montreal Cognitive Assessment; SCC, subgenual cingulate cortex; AVLT, Auditory verbal learning test; ITG, inferior temporal gyrus.

**Fig 6:**
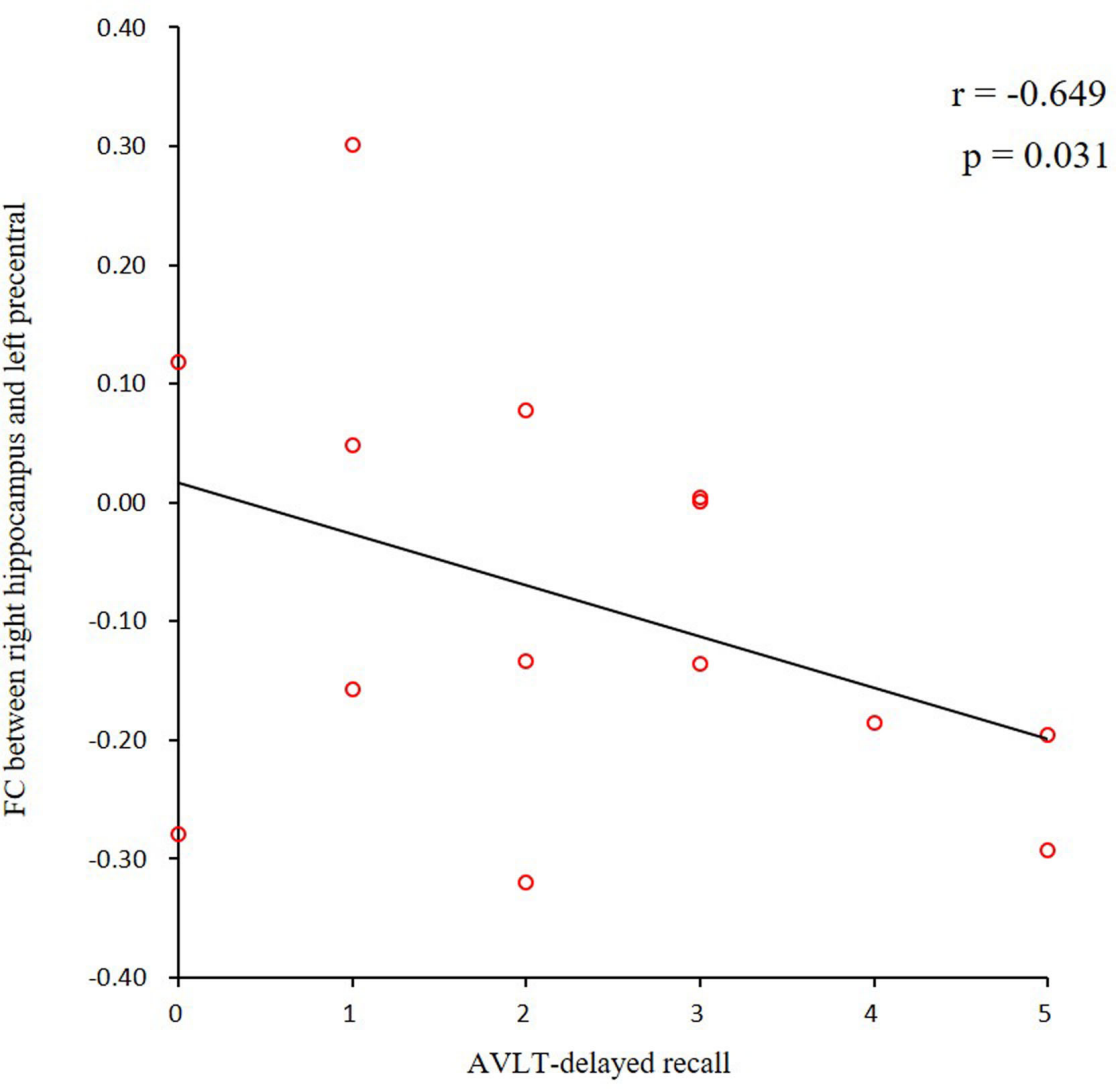
Correlations of clinical variables and hippocampus connectivity in AD patients. AVLT scores showed negative correlation with connectivity of the right hippocampus and left precentral gyrus (P<0.05). AD, Alzheimer’s disease; AVLT, Auditory verbal learning test.

In the AD group, we found positive correlations between the MMSE and MoCA scores and the ALFF values of the SCC. We also found negative correlations between the AVLT scores and the ALFF values of the hippocampus and the right ITG (**Fig 5**). In addition, we found a negative correlation between the AVLT scores and the connectivity of the right hippocampus and the left precentral gyrus (**Fig 6**).

## Discussion

Recent meta-analysis study investigated ten randomized controlled trials (RCTs) with AD treated by acupuncture [31]. Six trials showed that acupuncture was better than drugs on improving MMSE scores. Furthermore, there were evidence of 3 trails indicating that acupuncture plus donepezil was more effective than donepezil alone at improving the MMSE scale score. As a conclusion, clinical practice on AD confirmed the effect of acupuncture, which was better than that of drugs, and also, acupuncture can enhance the effect of drugs for treating AD. However, the mechanism of acupuncture on AD is not very clear and the evidence is insufficient. In the current study, we combined, for the first time, the ALFF and FC analyses to explore the effect of acupuncture on the AD patients. The following are the three main findings of the present study. First, multiple regions showed a decreased or increased ALFF, which was involved in some frontal and temporal regions, as well as cingulate cortex in the AD patients. Second, compared to the pre-acupuncture stage, several of the above regions showed an increased or decreased ALFF after acupuncture in the AD patients. Finally, hippocampal connectivity with the precentral gyrus showed enhancement after acupuncture in the AD patients.

At the baseline resting state, a decreased ALFF was revealed in the right SFG and the left postcentral gyrus in the AD patients relative to the HCs. The SFG is an important node of the DMN region. Previous studies have revealed abnormalities of DMN regions in AD patients, including cortical thinning [32], amyloid deposition [33], decreased intrinsic brain activity [12, 13] and disconnection of these regions [8]. The decreased brain activity in the SFG could lead to the cognitive decline and self-awareness dysfunctions in AD patients. In addition, the postcentral gyrus is the primary somatosensory cortex, which plays an important role in the sensorimotor network (SMN) regions. By analyzing the rs-fMRI data of 510 human subjects, Brier [34] found a significant functional abnormality in the SMN of the AD patients. Taken together with these findings, we speculated that subtle sensory impairment might exist in the AD patients due to dysfunction of the SMN.

Apart from the reduced spontaneous neuronal activities, we observed increased activity in the SCC, right MCC, right IFG, right hippocampus and the right ITG in the current study. These regions are located in the frontal and temporal regions, as well as in the cingulate cortex, most of which are cognition-related regions. For example, the cingulate cortex is an important component of the limbic system that has extensive anatomical connections with the regions of the prefrontal lobe, thalamus and striatum. It plays the role of a bridge or mediation between higher and lower cognitive levels in cognitive processing. The prefrontal cortex relays top-down cognitive control, and then integrates the information from the limbic system, such as cingulate cortex, to the higher-order cognitive center [35]. By using the resting-state fMRI method, Lin [36] found that higher IFG activity could protect memory performance and protect against the negative impact of AD-associated pathology. In addition, the hippocampus is one of the earliest pathological sites of AD and plays a crucial role in memory processes [15, 37]. Several studies have demonstrated increased activity in the hippocampus in AD patients [38, 39]. Finally, previous studies found that the strength of the ITG-hippocampus connectivity was positively associated with MMSE scores, indicating the cognitive function of the ITG [40]. Collectively, the exact role of the increased intrinsic activity of these regions is not clear, but it has been speculated that these regions increase their activities to compensate for the memory and cognitive decline of AD.

We selected the above seven regions as seeds to explore the ALFF changes in the AD patients between the pre-acupuncture and post-acupuncture stages. We found an increased ALFF in the left postcentral gyrus and the right SFG in the AD patients after acupuncture. We speculated that acupuncture could activate regions of the cerebrum that are responsible for sensation, memory and cognition in AD patients. In addition, we also found a decreased ALFF in the right IFG, right hippocampus and the MCC after acupuncture. These regions showed an increased ALFF in the AD patients at the baseline resting fMRI. We attribute the results to the dual-directional regulatory effects of acupuncture, which presented as activation in the decreased ALFF regions and deactivation in the increased ALFF regions in the AD patients.

We selected seven regions as seeds according to the ALFF analysis and performed seed-based FC analysis between the pre-acupuncture and post-acupuncture stages. After acupuncture, we found enhanced connectivity between the right hippocampus and the left precentral gyrus in the AD patients. As we all know, the hippocampus is responsible for memory and cognition and is one of the first regions of the brain that suffers damage in AD patients. Thus, assessing and monitoring hippocampal function are very useful for evaluation of AD. Using Morris water maze test, the aged mice used who received acupuncture showed significantly less cognitive deficit, which showed that acupuncture can prevent neuron loss in the hippocampus [41]. For the human subjects, the hippocampus atrophy correlates significantly with cognitive decline in patients with AD as well as MCI. In a previous longitudinal study of our group, we explored the correlation between strength of hippocampal connectivity and neuropsychological data and found the strength of the hippocampus connectivity showed significant positive correlation with MMSE scores in the MCI patients [40].In another longitudinal MCI study from other group, they also found positive correlation between the hippocampal connectivity and the episode memory scores [42]. In addition, some researchers found disruption of hippocampal functional connectivity in AD patients indicating of the cognitive impairment [14, 37]. In a recent acupuncture study related to the early stage of AD, the researcher detected increased hippocampal connectivity after acupuncture at acupoint KI3, which contributed to the cognitive improvement [43]. Collectively, based on the previous study, we speculated that increased hippocampal connectivity, which is induced by acupuncture in the current study, can enhance the information flow and result in improvement of cognitive function in AD patients.

We found positive correlations between the MMSE, MoCA scores and the ALFF values. However, we also found negative correlations between the AVLT scores and the ALFF values as well as hippocampal connectivity, we speculated that there might be some compensation to resist the cognitive decline: When the cognitive performances (AVLT) is impaired, some special regions may present over activation to keep the other cognitive function improvement. More study need to be performed in the future.

There are still some issues to be addressed. First, it will be more helpful to set a control state to compare real-needle acupuncture with sham acupuncture. Second, the current study was an immediate reaction from acupuncture, which didn’t focus on accumulating effects of acupuncture. Thus, longitudinal study of this acupuncture work is necessary. In the future, we will trace these subjects using different time points and explore the activity and connectivity changes and its influence on cognitive function in AD patients after the acupuncture. Third, in the future, a larger sample of fMRI data will be collected to test the current findings. In addition, we plan to combine more multimodal MRI techniques including metabolic, perfusion and diffusion methods to provide new evidence for the effect of acupuncture. Finally, recent studies have paid more attention to individuals at high risk for AD, such as amnestic mild cognitive impairments [44, 45] and ApoE-4 allele carriers [46] as well as subjective cognitive decline [47]. Exploring these populations would be helpful for searching potential therapeutic method at the early stage of the disease.

In conclusion, our findings provide evidence that acupuncture can induce significant regional alterations in AD patients, including increased and decreased spontaneous brain activity, as well as enhanced hippocampal connectivity. These findings may be helpful for deeper insight into the mechanisms of acupuncture and may provide a new method for the treatment of AD in the future.
